# Review on Bovine Tuberculosis: An Emerging Disease Associated with Multidrug-Resistant *Mycobacterium* Species

**DOI:** 10.3390/pathogens11070715

**Published:** 2022-06-21

**Authors:** Mohamed Borham, Atef Oreiby, Attia El-Gedawy, Yamen Hegazy, Hazim O. Khalifa, Magdy Al-Gaabary, Tetsuya Matsumoto

**Affiliations:** 1 Bacteriology Department, Animal Health Research Institute Matrouh Lab, Matrouh 51511, Egypt; mohameda.borham@yahoo.com; 2 Department of Animal Medicine (Infectious Diseases), Faculty of Veterinary Medicine, Kafrelsheikh University, Kafr El-Sheik 33516, Egypt; atef.ibrahim@vet.kfs.edu.eg (A.O.); yamen_hegazy@vet.kfs.edu.eg (Y.H.); magdy.elgabary@vet.kfs.edu.eg (M.A.-G.); 3 Bacteriology Department, Animal Health Research Institute, Giza 12618, Egypt; dr.attia31@yahoo.com; 4 Department of Infectious Diseases, Graduate School of Medicine, International University of Health and Welfare, Narita 286-0048, Japan; 5 Department of Pharmacology, Faculty of Veterinary Medicine, Kafrelsheikh University, Kafr El-Sheikh 33516, Egypt; 6 Antimicrobial Resistance Research Center, National Institute of Infectious Diseases, Higashimurayama, Tokyo 189-0002, Japan

**Keywords:** bovine tuberculosis, *Mycobacterium bovis*, multidrug resistance, animal health, diagnosis and treatment of tuberculosis

## Abstract

Bovine tuberculosis is a serious infectious disease affecting a wide range of domesticated and wild animals, representing a worldwide economic and public health burden. The disease is caused by *Mycobacterium* *bovis* and infrequently by other pathogenic mycobacteria. The problem of bovine tuberculosis is complicated when the infection is associated with multidrug and extensively drug resistant *M. bovis.* Many techniques are used for early diagnosis of bovine tuberculosis, either being antemortem or postmortem, each with its diagnostic merits as well as limitations. Antemortem techniques depend either on cellular or on humoral immune responses, while postmortem diagnosis depends on adequate visual inspection, palpation, and subsequent diagnostic procedures such as bacterial isolation, characteristic histopathology, and PCR to reach the final diagnosis. Recently, sequencing and bioinformatics tools have gained increasing importance for the diagnosis of bovine tuberculosis, including, but not limited to typing, detection of mutations, phylogenetic analysis, molecular epidemiology, and interactions occurring within the causative mycobacteria. Consequently, the current review includes consideration of bovine tuberculosis as a disease, conventional and recent diagnostic methods, and the emergence of MDR-*Mycobacterium* species.

## 1. History of Bovine Tuberculosis (bTB)

Tuberculosis affects a wide range of mammals and has been identified for thousands of years. When compared with other serious diseases, it is considered the biggest killer in the last 200 years [1]. In 1882, Koch discovered the tubercle bacillus of *Mycobacterium tuberculosis* (*Mtb*), the causative agent of human TB [2], whilst Smith in 1898, identified *M. bovis* as a different species from *Mtb* [3]. Bovine TB has a marked economic importance because of loss in productivity, morbidity, and mortality, in addition to the potential zoonotic threat [4]. The surveillance costs also have immense economic importance. Despite the disease representing a serious challenge in developing countries, regional disease foci that are not considered free of bTB continue to exist in the USA, Australia, and several European countries [5]. In addition, bTB is a massive concern in the UK and represents a major, ongoing problem for the British cattle industry [6].

## 2. Etiology of bTB

Among pathogenic mycobacteria, *M. bovis* and *Mtb* are the major causes of TB. They are highly pathogenic and may infect many animal species as well as humans [7]. Despite *M. bovis* being responsible for the vast majority of TB cases in cattle, it is not the exclusive cause of bTB [7]. Other mycobacteria were also isolated from cases of bTB such as *Mtb* and *M.* *caprae* [8]. *Mycobacterium caprae* is an essential factor in tuberculosis in ruminants [9].

*Mycobacteriumtuberculosis* was frequently isolated from diseased cattle in several countries such as Ethiopia, Nigeria, Egypt, South Africa, and India [1,7,10,11,12,13,14,15]. The disease caused by *M. bovis* is indistinguishable from that caused by *Mtb* and differentiation between them is very difficult to achieve either by clinical samples or cultivation [11]. Similarly, *Mtb* was reported to be isolated from bTB in buffaloes (*Bubalus bubalis*) [13], which paints a darker picture in the control of TB, especially in developing countries. In addition, tuberculosis is a severe threat to wildlife animals, including species under strict protection, such as European bison *(Bison bonasus)* or African elephants [16,17]. Also, bTB is a significant hazard in zoological gardens [18].

*Mycobacterium bovis* belongs to the Mycobacterium tuberculosis complex (MBTC) group that also comprises *Mtb*, *M. caprae*, *M. microti*, *M. africanum*, *M. canettii*, *M. pinnipedii*, *M. bovis* BCG, *M. leprae,* and the newly discovered *M*. *mungi*. These pathogens share an identical 16SrRNA sequence and up to 99.9% similarity at the nucleotide [19].

Mycobacteria are non-motile, non-capsulated, non-spore forming, with straight or slightly curved rods, are aerobic, oxidative, and of varying lengths (0.2–0.6 × 1.0–10.0 μm). Although they are cytochemically Gram-positive, the mycobacteria do not take up the dyes of the Gram-stain because the cell walls are rich in lipids, particularly mycolic acid. The mycobacteria are closely related to the genera Nocardia and Rhodococcus, which have a similar cell wall type [20].

Pathogenicity of mycobacteria is a multifactorial process that depends on the participation of several virulent factors of complex lipids in the cell wall, principally, in addition to protein and protein complexes. Mycobacteria possess a very thick cell wall containing a complex hydrophobic lipid; the cell wall core is composed of three attached molecules: peptidoglycan, arabinogalactan, and mycolic acid known as mycolylarabinogalactan–peptidoglycan complex. This complex is underneath a sheet like surface layer of glycolipids that made of superficial lipids as mycosides, cord factor, wax D, sulfolipid and sulfatides. This complex cell wall gives the pathogen some unique characteristics such as:

(a) Acid-fastness phenomenon attributed to high lipids content involving the mycolic acid hence, the name of acid-fast bacilli.
(b) Glycolipid complexes have some significance in the granuloma formation.
(c) Cords formation when grown on liquid media due to aggregation of complex lipids.
(d) The hydrophobic character of the lipid layer makes it impermeable to chemical agents and difficult to be stained by ordinary procedures. In addition, it causes poor penetration of nutrients, hence the slow growth of the organism and long incubation period of the disease.
(e) Resistance of mycobacteria to disinfectant agents, host’s immune system, and anti-tuberculous drugs [21,22,23].

The tubercle bacilli survival in the environment is extremely variable and ranges from a few days to 2 years depending on temperature, sunlight, and relative humidity. In general, tubercle bacilli survive best in cool, dark, and moist environments (buildings and transport vehicles) shaded from direct sunlight [24]. *Mycobacterium bovis* are inactivated by prolonged exposure to heat, direct sunlight, and dry conditions. They are killed by temperatures of 65 °C and above for at least 30 min, and UV light. In contrast, they are resistant to freezing for prolonged periods. Under ordinary temperatures, *M. bovis* can persist in slurry and soil for at least 6 months [25]. The high lipids content makes the mycobacteria resistant to several chemicals and disinfectants, but quaternary ammonium compounds, hexachlorophene and chlorhexidine have a bacteriostatic effect while formaldehyde vapor, chlorine compounds, 70% ethanol, hydrogen peroxide, alkaline glutaraldehyde, and 5% phenol have a bactericidal effect [23].

## 3. Risk Factors, Transmission, and Immunopathogenesis of bTB

Tuberculosis affects wildlife, livestock, and humans; hence, the extensive interfaces between these groups in developing countries is deemed a significant risk of transmission of the disease. Also, the movement of cattle from TB endemic areas was reported to be a great risk for TB breakdowns. Despite cattle being the main host of *M. bovis*, buffalo act as an important maintenance and reservoir host for the pathogen and maintain the disease transmission between different species. In addition, several wildlife species are considered reservoirs of TB, such as possum, wild boar, badger, and deer. Moreover, recent studies demonstrated the ability of members of MBTC, particularly *M. bovis*, to infect other livestock, such as goats, sheep, pigs, horses, camelids, and pets. Furthermore, alpaca has become a popular pet breed in many countries. There is a growing problem with tuberculosis in this species [26]. So, the possible role of alternative host species should be considered during the epidemiological investigation and design of TB eradication programs [27].

Broughan et al. [24] studied the effects of different risk factors on the transmission of TB and grouped them to: (A) Animal level risk factors which include genetics, breed, sex, age, reproductive status, milk yield, nutritional status, body condition and behavioral factors. (B) Herd level risk factors that include herd size, herd type, farm area, farm management, contact with neighboring herds, cattle movement, bTB history, and testing. (C) Environmental risk factors as landscape/soil type, weather, transmission through birds, invertebrates, and protozoa (experimental studies have demonstrated that *M. bovis* can survive in protozoa (*Acanthamoeba castellanii*), potentially facilitating transmission by extending the survival of the bacteria in the soil) [28]. (D) Wildlife reservoirs such as badgers, wild boar, and deer. The authors showed that many of these risk factors are interrelated, knowledge of the importance and identification of these risk factors is critical for understanding the infection dynamics and the development of more efficient and cost-effective approaches of disease control. 

Dejene et al. tested the correlation between different risk factors and the prevalence of bTB in cattle and reported that the older the age and the lower the body condition the higher the chance of a positive bTB test result, but sex, lactation status, and reproductive status were not correlated with bTB status. At herd level, the pastoral production systems with transhumant herds had a higher bTB prevalence than sedentary herds [29]. 

Transmission of *M. bovis* infection is generally by direct contact with tuberculous animals as the organism may be excreted in exhaled droplets, saliva, milk, urine, feaces (from both intestinal lesions and swallowed sputum from pulmonary lesions), semen, vaginal and uterine discharges, and discharges from open peripheral lymph nodes (LNs) [30,31]. The location and spectrum of the TB lesions are correlated with the route of infection. Infection mainly occurs through inhalation, sometimes by ingestion of contaminated pastures, food, and water, infrequently via transplacental, coital, or intramammary routes, and to a lesser degree through penetration of the agent through the broken skin. Thus, nasopharynx, lung and its associated LNs, oropharyngeal mucosa, and retropharyngeal LNs are the most common affected organs because the aerogenous and oropharyngeal routes are the most common pathway of infections [31,32].

Considering human to cattle transmission, Romha et al. refuted the hypothesis of transmission of *M. bovis* from human to cattle [7]. The authors mentioned that humans infected with *M. bovis* cannot transmit the infection to cattle. Contrarily, *Mtb* was frequently isolated from different animal species, justifying the possible transmission of the pathogen from human to animals [7]. The possible risk of this transmission could occur via aerosol infection from humans, especially in rural areas where animals live in close contact with tuberculous humans [33]. Also, the infection may occur through the practice of discharging of tobacco juice directly into the oral cavity ‘mouth-to-mouth’ of cattle as a common traditional anti-parasitic treatment in some areas in Ethiopia as identified by Ameni et al. [10]. Moreover, *Mtb* has been suggested to be transmitted to cattle through different routes, including ingestion of feed contaminated with infected sputum and/or urine from infected farmers [34].

Following the entry of mycobacteria through mucous membranes or into alveolar spaces, they are phagocytized by macrophages then through the lining of bronchioles, enter the circulation, and are carried to LNs, parenchyma of lungs, or other sites. The virulent tubercle bacilli possess the ability to escape being killed by macrophages through mycobacterial lipids such as lipoarabinomannan and mycobacterial proteins of the antigen 85 complex. Eventually, mycobacteria survive and multiply within the phagosomes, and destroy the phagocytes [22]. After the initial infection, the viable mycobacteria are transmitted through lymphatic capillary vessels to the draining LNs, where they establish a new infection focus. This dual infection is known as the primary complex [32]. 

The caseonecrotic granuloma is one of the hallmarks of TB, it is an attempt by the host to localize the disease process and to allow inflammatory and immune mechanisms to destroy bacilli and knowns as a tubercle. In addition, *Mycobacterium* has a direct action in granuloma formation through the secretion of virulence factors such as ESAT-6 [35]. After 10–14 days, the CMI responses develop, and the macrophages have increased capacity to kill the intracellular bacilli [22]. The granuloma formation passes through four stages; stage I (Initial), in which early lesions composed of accumulation of epithelioid macrophages, lymphocytes, neutrophils, and Langhans-type multinucleated giant cells. Stage II (Solid), as Stage I but has central infiltrates of neutrophils, lymphocytes, and a thin fibrous capsule. Stage III (necrotic), complete fibrous encapsulation and significant central necrosis are evident. Stage IV (necrotic and mineralized), multiple coalescing caseonecrotic granulomas with multicentric necrosis mineralization and thick fibrous encapsulation occur [30]. 

Tuberculosis spreads in the body by two stages, the primary complex and post primary dissemination. Tubercle of the primary complex in cattle is frequently detected in the lymph nodes of the head and thorax, involving the lung parenchyma only in 10–30%of cases [32]. The primary complex consists of the initial lesion at the point of entry and in the local lymph node, which may be missed at necropsy without careful examination. This small granuloma prevents further spread of the pathogen to the surrounding tissues and can remain arrested for a long time. Bacilli within the center of the lesion do not multiply but may remain dormant and the resulting latent infection may persist for years, this is known as the non-replicative persistence phase of infection [36]. In some animals, reactivation of the silenced pathogen occurs, tissue damage progresses, and the initially small granulomatous lesion becomes larger with time, caseated, necrotic, mineralized and fibrosed forming chronic or post primary dissemination. In other cases, when the immune response is ineffective, the generalization form results from hematogenous or lymphatic dissemination of the mycobacteria. The most common form of generalization is acute miliary tuberculosis. The generalization may be early generalization during the primary infection or may occur late in the post-primary phase or after reinfection. Some forms of generalization are extensive and cause diffuse caseation of lesions. These forms occur frequently in the lung and are usually called rupture forms, and it is assumed that the host’s CMI has waned [31,32]. The infection remains localized for months or years but can become generalized and the rate of progression of the disease is related to the challenge dose, cattle breed, as well as their immune and general health status [37]. 

As described above, the pathogenesis of bTB is complex and based on host immune responses to mycobacterial infection; infected animals can recover from bTB, progression of disease or persistence without progression [38]. 

The CMI response is the major host response to *Mycobacterium* spp. infection and mediated by T lymphocytes which release lymphokines that attract, immobilize, and activate additional blood-borne mononuclear cells at the sites where virulent mycobacteria, or their products exist. CD3^+^ and CD4^+^ T cells are the predominant lymphocyte subtype in granulomas of all stages. In addition, lower numbers of CD8^+^ T cells and γδ T cells can be found in Stages I–III. Many studies focused on the protective role attributed to CD4, CD8, γδ T cells and CD2; detection of changes in these cells considered as diagnostic markers for *Mycobacterium* spp. infection [30]. In addition, CMI develops the delayed-type hypersensitivity state in which lymphocytes and monocytes are directly or indirectly sensitive to tuberculin or related antigens [21].

## 4. Diagnosis of bTB

Diagnosis of bTB depends on traditional cellular immunity-based methods via tuberculin skin test (TST) and/or interferon gamma release assay (IGRA). In addition, antibody-based methods have recently emerged for diagnosis. Other methods, such as X-ray are also used to detect tuberculous lesions of tuberculosis and tuberculosis-similar diseases [39]. Furthermore, molecular based tools as mycobacterial interspersed repetitive units-variable number tandem repeats (MIRU-VNTR), spoligotyping, and restriction fragment length polymorphism (RFLP) are used for analysis of mycobacterial DNA for epidemiological investigation, determining the link between the new cases and prior outbreaks as well as the likely origin of infection. More recently, whole genome sequencing and rapid sequence analysis tools have been developed for a better comparison between MBTC isolates obtained from different species [40]. 

### 4.1. Field Diagnosis of bTB

#### 4.1.1. Tuberculin Test

Tuberculin skin test is the recommended standard procedure for ante-mortem diagnosis of bTB, which evaluates a delayed-type hypersensitivity reaction in sensitized cattle following an intradermal injection of purified protein derivate (PPD) tuberculin from mycobacteria, by measuring changes in skin thickness after 72 h from injection. In Egypt, as well as many other countries, PPD is injected in the mid-cervical region (cervical intradermal test), whilst in other countries as North America it is applied in the caudal fold of the tail (caudal fold test) [37]. The PPD injection stimulates the CMI and sensitizes T cells by prior infection which accumulate at the injection site and release lymphokines causing local vasodilatation, edema, fibrin deposition, aggregation of other inflammatory cells, and ultimately forming local skin swelling [41]. 

There are two commonly used types of the intradermal tuberculin test. The single intradermal (SID), in which PPD of *M. bovis* is used, despite its wide usage, high availability, and low costs, fails to differentiate between bTB infected cattle and others sensitized with *M. avium* complex or environmental mycobacteria. The second type is the comparative intradermal tuberculin test (CITT), in which both bovine and avian PPDs are injected simultaneously to increase the specificity of TST [42]. In Egypt, CITT is not used by general organization of veterinary services (GOVs) due to the lack of financial support. However, it is used only privately by owners of some herds [43].

OIE (2009) [44] define the standard TST procedures. (1) Clipping and shaving of the injected site. (2) Measuring a fold of skin of clipped area with a caliper. (3) A short needle with bevel edge in graduated tuberculin syringe is inserted obliquely into skin and the dose is injected. The recommended dose of bovine PPD must not be lower than 2000 IU. In cattle with diminished allergic sensitivity, a higher dose of PPD is needed, and in national eradication campaigns, doses of up to 5000 IU are recommended. (4) Palpating of a pea-like swelling at the injected area is a sign of correct intradermal injection. (5) The distance between two injections must be 12–15 cm distance. (6) The skin fold thickness is remeasured after 72 h where the same operator should measure the two readings. The positive bovine response is considered when the swelling is >4 mm, while <3 mm is considered negative, and between 3 and 4 mm is suspicious [45]. 

Byrne et al. found a strong positive relationship between the reaction size of injected PPDs and the severity of PM lesions and suggested that increased reaction size is indicative of progressive lesions. The authors also found that dairy breeds exhibited a greater reaction size compared to other non-dairy breeds [46].

Despite its wide use worldwide, TST has several drawbacks: (1) Complexity. (2) The interpretation is subjective and differs between operators. (3) False positive reactions when the animals were TST positive but there is no PM lesions nor positive culture, known as Non-Visible Lesions (NVL). They are given by cross reactivity to other non-virulent mycobacteria, avian TB, human TB, Johne’s disease (JD), or sensitized by other allergens as *Nocardia farcinicus* causing non-specific responses. Additionally, they occur in early stages of the disease, where TB granulomas are too small or infrequent to be seen during PM examination, or in infected animals during the latency in which infection with *M. bovis*, but without disease. (4) False negative reactions and those given during the late stage of infection, particularly in severe and generalized cases where the animals are non-responsive for TST and known as the State of Anergy. Also, the false negative reactions are given in early cases until 3–6 weeks post-infection which is known as the pre-allergic period. Further false negatives include animals desensitized by PPD administration during the preceding 8 to 60 days, old cattle, early post-partum cows (postparturient desensitization because of the general immunologic hyporeactivity), and low potency tuberculin, subcutaneous injection (rather than intradermal), or bacterial contamination of the tuberculin [24,31,47].

There are various factors affecting the sensitivity and specificity of TST, that range from 55–99%, such as potency, purity, dosage, and biological activity of the PPD, as well as the inoculation site, misreading of the results and the genetic background of the animal [42,47]. In addition, some co-infections affect the performance of TST particularly, the infection with *M.* *avium* subspecies *paratuberculosis* (MAP), the causative agent of Johne’s Disease, such cross-reactivity lead to misdiagnosis as MAP shares structural proteins and virulence factors with *M. bovis* [48]. In addition, co-infection with bovine viral diarrhea (BVD) virus has conflicting effects in previous studies; it is suggested to suppress the immunological response to PPD or cause rapid progression of bTB or even to have no significant effect [46]. Furthermore, co-infection with liver fluke, *Fasciola hepatica* and *Fasciola gigantica*, may affect the accuracy of bTB but the direction of the effect differs among studies [49]. It is suggested that liver fluke infection may suppress the *M. bovis* infection [46]. Howell et al. examined the evidence to determine the effect of liver fluke infection on four outcomes relevant to bTB diagnosis: TST, IGRA, lesion detection, and bacterial culture [49]. The study supported the hypothesis that liver-fluke-infected animals are likely to have a reduced response to both TST and IGRA tests and fewer bacteria recovered/cultured from their lesions. Furthermore, technical and procedural mistakes during TST application, related to measuring of skin thickness, storage and injection of PPD, and interpretation of test results, negatively affected the true detection of bTB [50].

Bovine PPD is a mixture of protein, carbohydrates, and lipids obtained from *M. bovis* AN5 and some of these components are present in nonpathogenic environmental mycobacteria [51]. Therefore, and for improvement sensitivity and specificity of TST, some trials for using some antigens instead of PPDs were performed; Parlane et al. displayed four mycobacterial proteins (ESAT-6, CFP10, Rv3615c, and Rv3020c) at high density on bacteria-produced polyester inclusions (biobeads) and concluded that their use should allow the development of a highly sensitive, specific, and cost-effective skin test for diagnosis of bTB [52].

Two approaches have been used globally at the herd level to overcome the limited sensitivity of TST in case of high prevalence settings. The first is the use of in vitro ancillary tests to maximize the detected number of infected animals and the other is the depopulation of the herds whose reactors are detected. Nevertheless, high costs and economic and social implications make whole herd depopulation difficult and inapplicable, particularly in endemic setting [53]. 

European Bison (*Bison bonasus*) is extremely sensitive to mycobacterial infection and its ongoing restitution requires the improvement of ante-mortem diagnostic methods such as TST and IGRA [54]. The authors used an intrapalpebral and recommended the use of the two tests in parallel instead of relying on one method alone.

#### 4.1.2. Ante-Mortem Examination (Clinical Signs) of bTB

The clinical signs of bTB vary depending on several factors: the sites of localization of infection, infectious dose, virulence, state of immune competence of the host, and external influences [55]. The incubation period ranges between 2 months, at minimum, and several years [56]. Most cattle that are infected do not develop clinical signs, but when present they are extremely variable and often nonspecific [57]. Because the disease is always progressive, there is the constant underlying toxemia, which causes weakness, debility, and the eventual death of the animal [31]. 

Pulmonary involvement is characterized by a chronic moist cough, thoracic abnormalities on auscultation heard especially in the advanced stages when much lung tissue has been destroyed, dyspnea with increased rate and depth of respiration become apparent. Lymph node enlargement coupled with chronic respiratory disease may result in a higher index of suspicion. Enlargement of bronchial LNs may cause dyspnea because of constriction of air passages, while retropharyngeal LN involvement may cause either respiratory signs and noisy breathing, or dysphagia and eructation. Meanwhile, the enlargement of the mediastinal LN is commonly associated with recurrent and then persistent ruminal tympany. Rarely, tuberculous ulcers of the small intestine can cause diarrhea. The reproductive disorders associated with bTB are uncommon; tuberculous metritis causing infertility sometimes with chronic purulent discharges, vaginitis, and rare cases of tuberculous orchitis. Tuberculous mastitis is of a great significance because of the public health danger, it is characterized by marked induration and hypertrophy, which usually develops first in the upper part of the udder, with supra-mammary LN enlargement. In case of miliary TB, some cows with extensive lesions are clinically normal, but in most cases progressive emaciation unassociated with other signs occurs. A capricious appetite and fluctuating temperature are also commonly associated with the disease [31,57].

#### 4.1.3. Postmortem Examination of bTB

Postmortem examination is a cornerstone for bTB control programs in endemic areas to detect the infection either in routinely slaughtered animals or in tuberculin test reactors [58]. Despite the wide availability of tests for the identification of *M. bovis* infection at the herd level, the diagnosis of bTB is often difficult due to the scarcity of diagnostic tests that fulfill all the essential criteria necessary for the identification of infected animals. It is noteworthy that almost the 20–30% of the new bTB cases are first diagnosed during postmortem inspection at the slaughterhouse in cattle intended for human consumption [58,59].

Bovine TB is characterized by formation of granulomatous nodules called tubercles which are circumscribed yellowish inflammatory nodules approximately 2–20 mm in diameter that are more or less encapsulated by connective tissue and often contain central caseous necrosis and mineralization. Tubercles are found in the LNs, particularly bronchial, retropharyngeal, and mediastinal nodes. They are also common in the lung, spleen, liver, heart, kidney, and surfaces of the body cavities [32,60]. Lesions may occasionally be found on pleural sac and in mammary tissues, some of them with cheese-like foci on cut surfaces. In general, some lesions appear as abscesses containing yellowish pus [61]. The pus has a variable color range from a characteristic creamy to orange, and consistencies from thick cream to crumbly cheese. In addition, lesion size may be microscopic or large enough to involve the greater part of the whole organ or tissue [23,31].

In lungs of extensively affected cases, diffuse pus is evident and may spread to cause suppurative bronchopneumonia as well as adhesion to the pleural cavity due to fibrinous inflammation. In addition, the presence of bronchopneumonia or hyperemia around pulmonary lesions is highly suggestive of active disease [31,62]. The chronic lesions become considerably enlarged, nodular, and contain thick, yellow to orange, caseous material, often calcified and surrounded by a thick fibrous capsule [31]. In disseminated cases, the miliary tuberculosis form is characterized by a large number of small grey to white yellowish caseous foci resembling millet seeds without clear-cut delimitations that are found throughout the lung and other organs. In addition, generalization to the serosal surfaces, especially the pleura, pericardium, or peritoneum, may occur and is characterized by multiple small tubercles of approximately 0.5 to 1 cm in diameter that resemble pearls. Lesions are sometimes found in the female genitalia but are rare in the male genitalia [32,60].

Interestingly, not all animals with bTB-like lesions would be detected at PM examination even with perfect examination techniques, because some lesions are invisible to the naked eye. The primary complex is most frequently located in the lower parts of the respiratory tract, 70% of these lesions may only be found during careful examination and dissection of the lungs into thin sections. Not all infected animals have lesions at the time of slaughter, as the existence of bTB lesions is directly related to the interplay between the host’s defense mechanism and mycobacterial virulence factors [32,58]. Moreover, it is important to point out other diseases whose lesions may resemble tubercles are common in other animal species [63,64,65]. Furthermore, most *Mycobacterium*-associated lesions are indistinguishable from mixed infections or lesions caused by other bacteria such as *Rhodococcus equi*. In cattle, pigs, and wild boars, *R. equi* infection is mainly associated with tuberculous-like lesions in lymph nodes. *R. equi* was isolated from cattle and American bison with purulent lesions suspected of *Mycobacterium* spp. infection. In pigs, *R. equi*, not the previously suspected *Mycobacterium* spp., is the primary causal agent of lymphadenitis. Furthermore, it was suggested that infection with *Mycobacterium* spp. could predispose farm and wild animals to *R. equi* infection [66]. Therefore, laboratory diagnosis is required to confirm suspected bTB cases.

### 4.2. Laboratory Diagnosis

#### 4.2.1. IFN-γ Release Assay (IGRA)

IFN-γ release assay is deemed the main ancillary test in bTB diagnosis and is usually used in parallel with TST [67]. It relies on in-vitro measurement of IFN-γ cytokine and has many advantages over other antemortem diagnostic tests, it being more sensitive, faster, and requiring one farm visit [68]. Additionally, it has one major advantage over TST, being able to detect more earlier infections than TST where a hypersensitivity reaction against PPD has developed between 3–6 weeks post-infection [51], while IGRA can detect the infection as early as 14 days after infection [69]. Thus, it can detect a substantial proportion of infected animals that escape detection by TST [47]. Moreover, it is also used to diagnose tuberculosis-similar diseases [70], and there are no limitations for retesting by IGRA because it, in contrast to TST, is an in vitro test.

Many previous studies reported that the prior administration of TST causes either a booster or a drop in IFN-γ release. These discrepancies are likely to be related to the variable conditions and type of skin test in each study [47,71]. Because TST may cause an apparent increase of IFN-γ production, it has been suggested to perform IGRA after 33 days to minimize its effect on the results [71]. In addition, Elsohaby et al. performed IGRA 45 days after TST [72]. In contrast, Clarke et al. studied the effect of timing of blood collection for IGRAs relative to the TST application in African buffaloes and recommended that collection of blood samples prior to or at the time of TST had a significance in the detection of a greater number of positive buffaloes than their collection three days after TST [73]. In the same line, parallel using of TST and IGRA maximized the detection of infected animal and was able to detect all infected buffaloes according to [74]. Additionally, De la Rua-Domenech et al. mentioned that blood samples can be taken as early as 3 days post-TST without affecting the results of the assay [47].

Abdellrazeq et al. pointed out, for the first time, cut-off criteria to optimize IGRA as a routine ancillary test for diagnosis of bTB in Egypt [43]. Also, Elsohaby et al. in Egypt, estimated the sensitivity and specificity of IGRA, PCR, and mycobacterial culture for detection of *M. bovis* in blood and milk, and recorded a higher sensitivity of IGRA than PCR and culture, and recommended the use of IGRA in the Egyptian bTB eradication program [72].

Despite its advantages, IGRA has high logistical demands (must be cultured within 24 h after blood sampling), and of high costs [75]. IGRA also has some limitations arising from using of PPD as antigen due to cross reactivity to environmental mycobacteria, leading several studies to develop multiple-antigen cocktails (Mb1762c, Mb2054c, Mb2057c, and Mb2660c) to increase the sensitivity of IGRA when supplemented to PPDs [76]. In addition, use of ESAT6 and CFP10 antigens instead of PPD in IGRA increased the ability to identify bTB infected animals and to distinguish them from NTM-exposed or BCG-vaccinated animals [45]. 

Novel biomarkers to distinguish between the healthy and infected animals are still urgently needed, particularly when TST and IGRA fail to detect the infection [42]. Hence, several biomarkers have been used in blood-based TB tests as interleukin (IL)-1β, IL-2, IL-17, IL-21, IL-13, IL-22, chemokine C-X-C motif ligand 9 (CXCL9), and CXCL10. Many of these cytokines are suggested for use as diagnostic biomarkers of *M. bovis* infection in cattle [77]. 

Both TST and IGRA detect the early bTB infection as they depend on the measurements of pathogen specific CMI responses [78]. At advanced stages of the disease, CMI responses decrease in parallel with increasing of the humoral immune response. Thus, TST and IGRA can give false negative results at the late stages [47]. Therefore, diagnosis of anergic animals, that show no response to CMI-based tests, is critical, because these animals could relapse and cause future outbreaks [79]. Thus, serological tests such as ELISA at the late stages of the disease are highlighted, especially with the adding of secreted proteins as MPB70 and MPB83 that are notably released by *M. bovis* in large amounts during the late stages of the disease [51,75]. 

#### 4.2.2. ELISA

Various factors affect the sensitivity and specificity of serological tests for diagnosis of bTB such as the stage of the disease, immune status of the animal, type of antigen used, and previous exposure to bovine tuberculin [80]. The sensitivity of antibodies-based ELISA is higher when evaluated in animals at later stages of the disease with gross lesions and in most infective animals [81]. In addition, they offer some advantages over CMI-testing, such as relative ease of sample collection, greater practicality, cost-effectiveness, and their ability to detect anergic animals [82]. On the other hand, others consider ELISA tests to be of a lower accuracy employ them less frequently than CMI-based tests for diagnosis of bTB due to their lower sensitivity and highly variable overall test performance [82,83]. Despite their precise role not being well understood, many factors participate in the variable performance of ELISA tests, such as geographical location, stage of infection and exposure to and diversity of NTMs [82].

TST is known to boost the antibody response; thus, the use of ELISA without skin tests would further reduce their sensitivity [83]. Testing of different sampling times is required to evaluate the best time to collect serum samples after PPD injection based on the increased sensitivity of the serological test [51]. Thus, several recent studies used ELISA tests to complement a prior TST; Casal et al. evaluated two ELISAs on sera collected prior to, and 3 days and 15 days after PPD injection, and reported the highest level of detected animals in samples collected after 15 days after TST, taking advantage of the anamnestic effect (increased serological response after performance of TST leading to an improvement of the sensitivity of the used technique) [81]. In addition, Fontana et al. validated a multi-antigen ELISA comprising five antigens (ESAT6, CFP10, PPD-B, MPB70, and MPB83) in sera collected 15–20 days after a single TST and demonstrated 74.2% and 94.9% of sensitivity and specificity, respectively [79]. Also, Souza et al. applied a recombinant chimera ELISA antigen of ESAT-6, MPB70, and MPB83 on sera obtained 7 days after a comparative TST, the sensitivity and specificity of the ELISA were 79.5%, and 75.5%, respectively [84]. More recently, Griffa et al. used an antigenic mixture from a total extract of the reference strain AN5 and were able to confirm the *M. bovis* infection of 83.7% of animals that were ELISA positive 15–17 days after a negative TST, by histopathology and PCR [85]. In the same study, the specificity was 95.95% and the authors suggested the detection of antibodies of *M. bovis* within weeks after TST as a rapid and inexpensive way to improve bTB control. On the other hand, Casal et al. took the serum samples before the injection of PPD and evaluated the sensitivity of three different ELISAs in addition to TST and two IGRA tests for the diagnosis of bTB in cattle and concluded that in vitro diagnostic techniques maximized the detection of bTB infected animals [86]. The authors suggested the parallel use of cellular and humoral-based tests in high prevalence setting conditions to accelerate bTB eradication because the antibodies-based tests significantly improved the sensitivity of cellular based tests up to 98.2%. Contrarily, McCallan et al. took the samples prior to TST to assess the utility of three serological tests, and their study reported that serological tests were of limited advantage when used in parallel with TST and IGRA, because the serological tests disclosed only about 3% of positive animals whilst TST and IGRA disclosed 13% and 40% of positive animals, respectively [87]. The authors suggested that the benefits of serological tests may be maximized if the samples are taken after TST.

Several studies highlighted the great significance of MPB83 protein as a diagnostic antigen in serological tests and recommended its use because of the highest and earlier response that was triggered against it [51]. Waters et al. reported that antibodies responses against MPB70 and MPB83 reach their peak 2 weeks after injection of PPD then begin to wane 1–2 months post TST, and can be further increased after re-injection of PPD [88]. 

IDDEX™ ELISA is a commercial kit recognized by OIE for detection of bTB infection in blood and milk serum samples depending on detection of antibodies against MPB70 and MPB83 antigens. It is used in several studies: Al-Fattli in Iraq used it as a screening test and reported a seroprevalence of 20.16% and 15.12% in blood and milk of lactating cows, respectively [89]. However, Soares Filho et al. recommended that IDDEX™ ELISA cannot be used as a single test for PM diagnosis of bTB because of its low sensitivity despite the test being able to detect eight positive samples that were negative on RT-PCR and culture [80]. The authors collected blood and obtained serum aliquots from the brachial vein venous blood of the half carcass from which the respective tissue and organ samples were obtained. Trost et al. reported a wide variation in sensitivity of IDDEX™ ELISA by geographical distribution, where it was 9%, 45%, and 77% from bovine serum samples in Mexico, the United States, and the United Kingdom, respectively [90]. The authors compared the sequence variation in four genes, mpb70, mpb83, sigK, and rskA, of 455 *M. bovis* strains from the three countries in an attempt to explain these geographical disparities. However, the study concluded that sequence variation in these genes does not explain the variation in sensitivity.

Other serological tests are used in several studies, such as rapid lateral-flow test for detection of antibodies against *M. bovis mpb70* antigen. Elsohaby et al. used it in conjugation with TB-Feron test (a type of IGRA test) as ancillary tests and both were able to reduce a significant number of false positive TST slaughtered cows [91].

All the previous tests, TST, IGRA, and ELISA, have wide various limitations related to their sensitivity and specificity, thus, parallel use of more than one test and focusing on finding new cocktail mycobacterial antigens offer substantial advantages for maximizing the detection of infected animals [45,51,76]. 

#### 4.2.3. Mycobacterial Culture

Isolation and identification of the mycobacteria is still the international gold standard test in diagnosis of bTB [44]. It can take up to three months due to the slow growth rate of MBTC [92]. 

The most frequently media used for mycobacterial growth are Lowenstein–Jensen buffered egg potato medium, Middle brook 7H10, Middle brook 7H11, and Dubos Oleic-Albumin agar. Unlike *Mtb* and *M. avium* that grow well on glycerol containing media and known as eugenic, *M. bovis* has a sparse thin growth on them and is called dysgenic, but it grows well on pyruvate-containing media without glycerol [93]. This dysgenic growth of *M. bovis* in the presence of glycerol is due to lack of pyruvate kinase enzyme [94]. The growth of moist, white, flat, and friable colonies is indicative for the primary cultures of *M. bovis* on Lowenstein–Jensen slants [95]. Zihel–Neelsen staining should be performed to confirm the presence of acid-fast bacilli. Despite being rapid and sensitive, Zihel–Neelsen lacks specificity and cannot differentiate between members of MBTC [96]. 

A range of pre-culture treatments, such as decontamination, homogenization, and concentration are conducted before inoculation on the media to facilitate the recovery of *M. bovis* [97]. The most traditional decontamination method used to isolate *M. bovis* from bovine tissues is the Petroff method in which 4% NaOH solution is used as a decontaminant (OIE 2014).

Mohamed et al., compared between BACTEC MGIT 960TM as a fully automated liquid-medium system and the conventional culture using Lowenstein–Jensen media for isolation of mycobacteria and found that automated system was more sensitive, faster, and revealed a higher recovery rate of mycobacteria than Lowenstein–Jensen media [96].

Issa et al. in Brazil, compared three decontaminants used in mycobacterial decontamination: 2% sodium hydroxide (NaOH), 0.75% hexadecyl pyridinium chloride (HPC), and 5% sulphuric acid (H_2_SO_4_) [98]. In addition, they evaluated four mycobacterial media: Middlebrook 7H11 with additives and OADC (oleic acid, albumin, dextrose, and catalase) supplement A (7H11-A), Middlebrook 7H11 with another supplement trademark (7H11-B), tuberculosis blood agar (B83), and Stonebrink’s medium. The authors found that using 5% H_2_SO_4_ and inoculation of Middlebrook (7H11-A) was the best strategy for the primary isolation of *M. bovis.*

For achieving the best results, Soares Filho et al. recommended two sampling protocols for both PCR and isolation [80]. For PCR, the tissue was collected from the center of the caseous lesion, where more genetic material and fewer viable bacteria are expected to be found whilst, for isolation, collection was done at the border between the healthy tissue and the lesion, where more viable bacteria, but less bacterial genetic material, would be expected.

Despite mycobacterial culture usually being regarded as the golden standard, it is time consuming and prolonged for several months, risky, laborious, with a high level of tissue sample contamination and decreased number of viable mycobacteria due to the decontamination methods [80,97,98]. In addition, Albernaz et al. reported a low sensitivity of bacterial culture and stated that it is not recommended as a routine complementary test for diagnosis of bTB in buffaloes [99]. The major limitation of mycobacterial culture is being confined to PM lesion samples, some studies suggest the use of nasal swabs as an alternative method [75]. However, Mayer et al. stated that RT-PCR from nasal swabs is not suitable for in vivo diagnosis of bTB [100].

#### 4.2.4. Histopathology as a Diagnostic Method of bTB

Canal et al. described the characteristic histopathological pictures of tuberculous lesions that were classified microscopically into four stages (stage I, II, III, and IV) [101]. Stage I, in which irregular epithelioid macrophages, dispersed lymphocytes, and few Langhans-type multinucleated giant cells are displayed. Stage II granulomas exhibited limited necrosis with neutrophils, lymphocytes, macrophages in addition to few fibroblasts and Langhans-type cells. Stage III granulomas exhibited epithelioid and Langhans-type giant cells in the peripheral areas of the central caseous necrosis, with central calcification. Near the fibrous capsule, the inflammatory cell population consisted of macrophages, lymphocytes, and scattered neutrophils. Stage IV granulomas exhibited mostly necrosis and mineralization. A large fibrous capsule was evident, and this capsule shaped an irregular area of large necrosis and mineralization. Evidence of thick encapsulated lesions is suggestive for lower dissemination and an active anti *M. bovis* immune response.

Despite high sensitivity, histopathology lacks specificity; McKinley et al. described a histopathological profile with encapsulation of granuloma, presence of Langhan’s cells, sometimes in association with epithelioid cells, lymphocytes, or neutrophils in tuberculous-like lesions [102]. However, neither *M. bovis* nor member of MBTC were detected either by molecular methods or cultivation over 3 months, but NTM and Actinomycetales were identified in the lesions. 

#### 4.2.5. Molecular Diagnosis

PCR techniques are widely used for the diagnosis of bTB and have several advantages; they are quick, applied within a few hours which means rapid diagnosis and efficient control, overcome the lack of specificity of other traditional tests such as histopathology, and are able to identify the mycobacteria either from culture or clinical specimens [68,103]. Moreover, several PCR techniques targeting numerous genes and regions can be used for differentiation between members of MBTC, such as ESAT-6 and CFP-10, which are protein products of *esxA* and *esxB* genes, respectively [104], *atp*E and *lpq*T [1], regions of difference (RD 1, 4, 9, 12) [1,105], RvD1-Rv2031c [76], and insertion sequences (IS) as *IS1081* [80] and *IS6110*. Numerous studies targeted the insertion sequence region *IS6110* as it is found in all members of MBTC [103,104,106].

Thacker et al. reported a high specificity of TaqMan Real-Time PCR targeting *IS6110* gene as it was able to detect 5 pg/μL of *M. bovis* specific DNA or even smaller quantities in tissue samples [106]. Despite Soares Filho et al. reporting a low sensitivity of the PCR technique as a PM diagnostic method of suggestive tuberculous lesions, the authors recommended the usage of PCR in situations of high prevalence or in parallel with other tests such as ELISA because it is a quick, safer, and relatively less expensive technique [80]. On the contrary, Algammal et al. reported a higher sensitivity of PCR, over 85%, compared to other PM diagnostic methods [68]. 

Several other reports detected lower sensitivity and specificity of PCR. The sensitivity of the molecular studies varies from 50% to more than 80% depending on the study and the employed methods [107]. The complex mycobacterial cell wall, presence of the bacterial cell within granulomas and the presence of PCR inhibitors and subsequent failure of DNA extraction are obstacles facing the PCR diagnosis of MBTC organisms [108,109]. 

The varying results of PCR technique performance are mainly because of technical differences in the setting up of assays, particularly during DNA extraction from lesions [80,109], and their sensitivity is conditional on sensitivity of necropsy and volume of DNA [110]. Further, contamination of the PCR reaction and the presence of environmental bacteria can prompt false positives and cause insufficient specificity [106]. Differences of the PCR primers used [83] and the presence of inhibitory substances in samples or reagents can also cause reduced sensitivity [109].

There is a significant variation of sensitivity of PCR assays related to the DNA extraction method used and there is not a definitive view for the best method of DNA extraction from bovine tissues [111]. Hence, several studies compared DNA extraction protocols; Ikuta et al. compared three protocols and reported 46.6%, 50%, and 100% positive samples of the three protocols from the same samples [109]. In addition, Moura et al. evaluated nine DNA extraction methods, using nine commercial kits, reported various results between them and concluded that DNA extraction kit deeply influences the diagnostic sensitivity of bTB in bovine tissue samples [112]. 

Despite their advantages, PCR techniques have several limitations; limited to the PM diagnosis [80], not specific for pathogen identification, being restricted only to members of MBTC or *M. avium* complexes [113]. In addition, reduced sensitivity of some PCR assays due to inhibitory substances in samples or reagents, during DNA extraction, as well as lower amounts of DNA [23,109]. Helmy et al. reported that RT-PCR was more sensitive and specific than conventional and multiplex PCR, less manipulated and possessed low risk of cross-contamination [12]. In addition, Algammal et al. reported that RT-PCR is the most sensitive rapid diagnostic test for detection of *M. bovis* from tissues and provides higher positive values than culturing [68]. However, other studies confirmed the usefulness of combination between RT-PCR and conventional PCR for rapid identification and discrimination between members of MBTC [1].

Besides tissue samples, many PCR assays had been performed on blood and milk samples such as the Mataragka et al. study, which illustrated that PCR can be used as an early and sensitive indicator method to detect infection in pooled milk samples collected from the aged animals of a dairy farm, which could support TST monitoring and improve bTB control [114]. In addition, Brahma et al. reported a similar sensitivity of PCR targeting CFP-10 protein to that of IGRA and concluded that it may be used as a fast, alternative method for bTB diagnosis from blood samples [4]. Elsohaby et al. reported a difference between the PCR estimates of *M. bovis* in both blood and milk in dairy cattle, where the sensitivity of PCR conducted on blood and milk samples was 53–95% and 1–60% while its specificity was 95–100% and 43–99% for each sample, respectively [72]. The authors attributed these differences to the type of sampling, that largely affect the PCR sensitivity and specificity estimates. However, further studies about bacteremia and the time of dissemination of *M. bovis* in blood stream is needed to detect the proper sampling time because most of TST and IGRA reactor animals failed to be detected by PCR in blood samples [4]. Despite the high risk of disseminated infection of *M. bovis*, bacteremia has been assumed to be rare in cattle [115].

In the light of the aforementioned, bTB lacks a definitive gold standard diagnostic test [80] since no single diagnostic method is able to detect all infected animals [113]. In addition, several classical tests based on growth, phenotypic, and biochemical properties had been assayed to discriminate between members of MBTC [47]. However, these tests were inaccurate, slow, time consuming, cumbersome, and cannot be performed in any laboratory [75]. Hence, the usage of advanced molecular diagnostic methods has been highlighted in recent years, especially because all members of MBTC share about 99.9% of nucleotide identity [116]. Spoligotyping and MIRU-VNTR profiling have been widely used as molecular typing methods of MBTC members [117,118]. However, due to a low discriminatory power, these methods have limitations for phylogenetic studies so, the advances in the high-throughput sequencing technology become urgent over the last decade [119].

Whole Genome Sequencing (WGS) provides new insights into dynamics of disease transmission, host-pathogen interactions, and comparative analysis that elucidates key differences between the animal and human mycobacteria [75,120]. Abdelaal et al. performed WGS on Egyptian *M. bovis* isolates from Nile Delta and reported a predominance of isolates which were closely related to clonal complex (European 2). In addition, the authors reported two isolates belonged to *M. bovis* BCG group that are rarely isolated from animals [120].

Clarke et al. investigated an in-field sampling technique for rapid, safe detection of *M. bovis* in buffalo tissues [121]. The authors recommended the use of PrimeStore^®^ Molecular Transport Medium, in combination with Xpert^®^ MTB/RIF Ultra assay (an automated cartridge-based semi-quantitative nested RT-PCR assay that detects DNA of MBTC and rifampicin (RIF) resistance in clinical specimens) as a safe and rapid PM screening test for *M. bovis* in buffaloes. In humans, Xpert MTB/RIF has recently become a significant breakthrough in TB diagnosis [122]. This test can detect MBTC DNA and mutations associated with antitubercular drug resistance.

Kapalamula et al. developed a loop-mediated isothermal amplification (LAMP) method for specific identification of *M. bovis*. This LAMP method was able to detect *M. bovis* within 40 min following incubation and results could be read with the naked eye following development of a color change [123]. The authors recommended the LAMP as a rapid and low-cost method for detection and surveillance of *M*. *bovis* infection in cattle and humans in resource-limited, endemic areas.

Additional tools for diagnosing TB, such as lymph node biopsy and tracheobronchial aspirates, have been successfully implemented in wildlife ruminants. Didkowska et al. used tracheobronchial aspirates and ultrasound-guided biopsies for diagnosis of TB in European bison [124]. In addition, Didkowska et al. used endoscopy (bronchoscopy) as an additional tool for diagnosing tuberculosis in European bison, especially in highly valuable animals, and to assess the stage of the disease [125]. In human, sputum samples are widely used for diagnosing pulmonary tuberculosis [126]. 

## 5. In Silico Analysis

Bioinformatics is the science of analyzing and managing biological data using computational tools and algorithms for expanding the use of biological, medical, behavioral, or health data. It uses genetic databases and biological samples and provides a time-saving and cost-effective tool to obtain an important data on gene and protein levels not easily obtainable by other techniques [127]. Research in bioinformatics includes algorithms designed for storage, retrieval, and data analysis. Bioinformatics is a fast-developing field of science combining biology, information engineering, computer science, mathematics, and statistics to examine and understand biological phenomena. It has practical applications in specific areas such as molecular biology and medical disease diagnosis [128].

In silico analysis tools have been highlighted for analyzing the sequenced genome of mycobacteria, studying the interaction between the gene mutations and anti-tuberculous drug resistance, and for prediction of the drug resistance particularly in humans [118,129]. A recent study in France used bioinformatics analysis in human TB and predicted that 112 *Mtb* isolates were scattered among 4 lineages and 25 sub lineages [130]. In addition, the analysis predicted 8%, 4.4%, and 1.7% of rifampicin-resistant (RR), multi-drug resistance (MDR) and extensively drug-resistant (XDR) isolates, respectively. In addition, Agarwal et al. in India used several bioinformatics tools for studying the structure and function and detection of mutations of folE protein of Rv3609c gene in *Mtb* H37Rv strain [131]. The study has also predicted the functional regions and interacting partners involved with folE protein and recommended its use for drug targeting after experimental analysis of this protein. Furthermore, Sun et al. in China used bioinformatics analysis to identify novel biomarkers of pulmonary TB in humans [132]. The study helped to estimate pulmonary TB prognosis and provided a probe into targeted molecular treatment. Keikha et al. in Iran used in silico tools for designing a multi-epitope ESAT-6: Ag85b: Fcγ2a fusion protein as a novel candidate for a TB vaccine [133]. More recently, Bibi et al. in China applied advanced computational techniques to develop a universal TB vaccine [134]. The authors used bioinformatics tools to define tuberculosis novel multi-epitope subunit vaccine, which is highly immunogenic and has appropriate properties to be a carrier vaccine. In addition, epitope prediction tools were used to analyze multiple B cell and T cell epitopes to enhance the vaccine’s immunogenicity. Jia et al. in China used bioinformatics for comparative genomic analysis of 12 MBTC strains, in between *M. bovis* and *Mtb* which were obtained from various hosts including humans and cattle [135]. The analysis provided insight into dissimilarities between intraspecific groups differing in host association, virulence, and epitope diversity, and facilitated the development of potential molecular targets for the prevention and treatment of TB. Perea Razo et al. in Mexico used in silico tools to detect spoligotypes through analysis of WGS of 322 *M*. *bovis* isolates from different sources; dairy and beef cattle, as well as humans [136]. Palaniyandi et al. used bioinformatics for comparative analysis of WGS of *Mtb* isolates from cattle and their attendants (People near cattle) in South India [137]. The study examined the relatedness of *Mtb* from cattle and their handlers and detected three isolate pairs that were highly related, of which two pairs were from handlers and one was from cattle, suggesting that *Mtb* transmission occurred between handlers, either directly or through an intermediate host. Also, it was suggested that *Mtb* was transmitted between two cattle who shared a highly related strain. Assal in Canada used bioinformatics tools for the prediction of extracellular proteins from *M. bovis* genome sequences and identified 96 protein candidates. In addition, the proteomics analysis identified additional 92 protein candidates secreted by *M. bovis* [138].

In Egypt, studies that used bioinformatics tools regarding bTB are scarce; Abdelaal et al. in Nile Delta of Egypt used in silico analysis to detect lineages and spoligotypes of *M. bovis* isolates. Although several studies on the bioinformatics analysis of human TB and its related mycobacterial isolates are existing [120]. However, such type of analysis on bTB is still limited worldwide, and is nearly absent in Egypt, except for one study by Abdelaal et al. [120]. Recently, Borham et al. in Egypt used bioinformatics tools for prediction of mutations, nucleotide polymorphisms, lineages, drug resistance, and protein–protein interactions (PPI) of ten *Mycobacterium* sequenced strains [139]. Future in silico analysis studies are strongly required for bTB and its related isolates in Egypt as well as worldwide to understand many mysterious aspects of bTB.

## 6. Zoonotic Importance of bTB

The infection in humans, is primarily acquired following consumption of contaminated unpasteurized dairy products or, to a lesser extent, undercooked meat, causing extrapulmonary TB cases [140,141]. Furthermore, it occurs through direct contact with infected animals (airborne transmission) causing pulmonary TB particularly in farmers, veterinarians, and slaughterhouses workers [142,143]. Ibrahim et al. clarified that the identification of both *M. bovis* and *Mtb* in slaughtered cattle, suggesting anthropozoonosis (transmission between humans and cattle) as a public health concern [15].

Zoonotic TB poses a significant hazard; in 2016, there were 147,000 new cases and 12,500 deaths in people worldwide [144]. The true burden of the disease is due to a lack of routine surveillance data from most countries, resulting in the underestimation of the zoonotic importance of tuberculosis. Zoonotic tuberculosis caused by *Mycobacterium bovis* is also underestimated in humans because the commonly used diagnostic techniques do not allow identification of the pathogen at species level [145,146]. Zoonotic TB creates challenges for the treatment and recovery of patients because *M. bovis* is naturally resistant to pyrazinamide (PZA), which is one of the key drugs used in the treatment of TB [144]. Zoonotic TB remains an important unaddressed global problem, especially with evidence of new zoonotic mycobacterial strains in Southeast Asia and Africa (e.g., *M. orygis*). *M. bovis* is considered the proxy for zoonotic TB, it is responsible for up to 37.7% of all human TB cases in Africa [147]. However, other mycobacteria present in animals and the environment can cause zoonotic TB, these include *M. canetti*, *M. caprae*, *M. microti*, *M. pinnipedii*, *M. mungi*, and *M. orygis* [148]. According to the 2020 WHO Global Tuberculosis Report, out of 10 million with new active TB, 140,000 (1.4%) are estimated to be new cases of zoonotic TB [149]. The highest numbers were reported from Africa and Southeast Asia. Southeast Asia comprises almost 44% of the global TB burden [149]. The existence of multiple carriers magnifies the problem; deer, antelopes buffalo, wild boar, brushtail possums, bison, goats, camels, alpaca, llama, pigs, European badgers, dogs, and cats in addition to the primary reservoir cattle [148]. There are several risk factors for *M. bovis* transmission that govern bTB epidemiology and transmission to humans. These factors include demographic factors (e.g., number of family members, age), feeding habits, people living in close contact with their animals, socio-economic status, illiteracy (lack of knowledge of zoonotic TB), the wildlife–livestock–human interface is particularly relevant in Sub-Saharan Africa [150].

## 7. Multidrug and Extensively Drug-Resistant TB and bTB

The development and emergence of multidrug resistance in animals has gained worldwide attention owing to the possibility of pathogen transmission to humans [151,152,153,154]. Multidrug-resistant TB (MDR-TB) is caused by TB bacteria that are resistant to at least two most potent TB drugs including isoniazid (INH) and rifampin (RIF) which are used to treat all patients with TB disease (CDC, https://www.cdc.gov/tb/, accessed on 10 May 2022). Extensively drug-resistant tuberculosis (XDR-TB) is a rare type of MDR-TB in which at least four of the most powerful and core anti-TB drugs cannot act against bacterial activity. These drugs include INH and RIF, levofloxacin/moxifloxacin, and at least one second-line injectable drug such as capreomycin, amikacin, or kanamycin. Misuse or mismanagement are the major causes of the development of resistance to anti-TB drugs (WHO, https://www.who.int/news-room/questions-and-answers/item/tuberculosis-multidrug-resistant-tuberculosis-(mdr-tb), accessed on 10 May 2022). According to a WHO report (2019), in 2018 alone, there were approximately half a million (range, 417,000–556,000) new cases of RR-TB, of which 78% had MDR-TB. Moreover, among cases of MDR-TB in 2018, 6.2% were estimated to have XDR-TB [155].

The problem of MDR-TB was recently exaggerated by its emergence in animals. Tuberculosis is most commonly treated with pyrazinamide (PZA), INH, RIF, and ethambutol, followed by streptomycin and kanamycin [156]. Previous reports confirmed that *M. bovis* is known to be naturally resistant to PZA owing to the presence of C→G point mutation in the *pncA* gene, at nucleotide 169, which confers natural resistance to PZA. Different reports documented an emerging trend in the incidence of INH-resistant *M. bovis* in Ireland, as well as an outbreak caused by multidrug-resistant *M. bovis* strains—resistance, at least to RIF and INH—is also documented in other literature with a dramatic impact [156]. Furthermore, Vazquez-Chacon et al. described the characterization of six human *M. bovis* clinical isolates including three MDR strains [157]. The authors identified mutation in both *katG* and *rpoB* genes that conferring resistance to first line antibiotics in the MDR *M. bovis* strains. Furthermore, they confirmed the absence of genetic relationship between human *M. bovis* strains or between human and bovine strains. Recently, Abdelsadek et al. in Egypt tested 135 MBTC isolates obtained from both infected cattle and veterinarians or workers in contact with these animals [158]. The study reported high resistance for both PZA and INH of 78.5% and 59.3%, respectively. In addition, MBTC isolates were highly resistant to the second-line drugs; KAN and AMK in a percentage of 82.3% and 80.7%, respectively. Borham et al. reported one *Mtb* strain that was MDR to RIF, INH, and streptomycin, this was the first report of multidrug resistant (MDR)-*Mtb* originating from buffaloes [139]. In addition, the authors reported seven *M. bovis* strains that were resistant to ethambutol and ethionamide. Anne et al. emphasized the importance of more research on MDR strains of *Mtb* isolates from bovines particularly in endemic areas due to the high possibility of reverse zoonosis [159]. Hence, the well-documented resistance profile will provide useful empirical data for better treatment and management of TB and make the epidemiological surveys simpler [160].

## 8. Treatment, Prevention, and Control of bTB

Bovine TB is rarely treated in domestic livestock except in rare cases of valuable animals as zoological exhibits. Anti-tuberculous first-line chemotherapies that have the greatest activity include INH, RIF, PZA, ethambutol (EMB), and streptomycin (SM). Second line drugs include capreomycin (CAP), ethionamide (ETH), cycloserine, and thioacetazone [40]. Primary control strategies of bTB depend on testing of infected animals by TST and/or IGRA, isolation of the infected animals within the infected herds, routine slaughter surveillance, slaughtering the infected animals, movement restriction of the affected herds, and removing the TB test-positive animals (reactors). In addition, movement/border testing policies, thorough epidemiological investigation of reported cases, and certification of a negative TB test(s) prior to entry of animals to countries, particularly for animals emerging from bTB-affected regions, are important procedures for controlling bTB [40].

The control system for identification of bTB comprises TST, abattoir surveillance and PM confirmation. There is a dynamic link between these three arms and they could not be considered independent because the deficiencies in TST will manifest in an increased number of lesions at abattoirs. Likewise, missed lesioned animals at slaughterhouses will delay the detection of undisclosed transmission within herds [102].

Complete eradication of bTB has not been achieved in any country due to the presence of major problems facing the eradication: (1) Breakdowns: disclosure of bTB affected animals either through detection of TST-positive animals after a successive number of free tests within herds or through confirmation of the infection within abattoirs by bacteriological culture. Breakdowns are caused by anergic carrier or a break in the security of the herd or infection from animals newly introduced to free herds. (2) Non-Visible Reactors (NVL) that create administrative and public relations difficulties. (3) Presence of wildlife reservoirs. (4) Large herds: as in North America and South America where cattle are run under very extensive conditions on large ranches or stations. Also, in intensive dairies where the complete depopulation of herds cannot be performed [31,102]. Positive TST results are sometimes neglected in countries with rare TB cases. It is unacceptable, but some vets assume that the positive result for bovine tuberculin is a reaction against *M. avium* and do not follow the mandatory CITT procedure, in which both bovine and avian PPDs tuberculin are tested for.

Vaccination against bTB is rarely used for controlling the disease in areas with official control programs because BCG can induce a cellular immune response producing interference with the testing strategies using PPD as antigen (TST and IGRA). Lack of the available diagnostic tests to differentiate between infected and vaccinated animals is the main impediment [161]. In addition, the live attenuated BCG vaccine has some problems, such as uncertain stability in natural conditions and its possible survival in tissues, secretions, and environment [162]. However, some recent experimental trials with bacillus Calmette Guerin (BCG) in cattle demonstrated that it was particularly effective when administered to neonates [163].

Chandran et al. demonstrated a novel BCG vaccine to protect animals against bTB. The authors also demonstrated a novel skin test for differentiating infected from vaccinated animals (DIVA) that will detect bTB [164]. Nevertheless, several trials have been used in many animal models using heat-inactivated and formalin-killed vaccines with variable results, and great challenges still remain toward production of effective, safe, and easily-implemented vaccines in domestic animals [162]. Being zoonotic and causing a severe public health hazard, many prominent organizations as OIE, WHO, FAO endorsed the One Health approach of TB to comprehensively address the challenges at the animal–human interface [165]. 

## 9. Bovine TB in African Countries in Special Regard to Egypt

Bovine tuberculosis stills representing a big challenge in developing countries, especially in Africa due to interactions between people, livestock transhumance, and wildlife. In addition, deficiencies in preventive and/or control measures, poor sanitation, veterinary and slaughterhouse services, and lack of political measures are the main causes of persistence of the disease in Africa [166]. 

Regarding Egypt, the first record of bTB was by Piot Bey in 1917 [167]. In 1920, the overall rate of infection in the cattle and buffalo population was estimated at 2–9% by tuberculin testing. This prevalence dropped to 2.6% in 1985 because of the establishment of a national program which started in 1981 [168]. Since the 1990s, and according to the recent official reports of the GOVs, the annual proportion of bTB-infected cattle has increased relative to the importation of live animals from countries where bTB is prevalent. The disease is extensively concentrated in the Egyptian Nile Delta and Valley relative to elsewhere in the country [43].

There are several challenges facing bTB control in Egypt; lack of financial support is a big challenge that prevents covering the entire cattle population, which estimated to be 4.9 million cows and 4.1 million buffalos, during the national control program testing by SID only a quarter of this population is tested. In addition, the single comparative cervical tuberculin test is not practiced by the general organization of veterinary services due to lack of financial support. Most abattoirs in Egypt do not have diagnostic facilities for rapid confirmation of grossly detected bTB lesions. Import of live animals from some endemic countries is recognized as a potential source of bTB transmission into Egypt. Moreover, the majority of cattle populations are raised by smallholders individually or with other animals such as sheep, goats, and probably camels, horses, dogs, and cats at rural areas in both Middle Delta and Middle Egypt regions, where they share the same pasture and the same shelter [43,168,169]. Bovine TB is responsible for condemnation of a significant amount of inspected meat and viscera in Egypt. Elmonir & Ramadan estimated the monetary loss due to condemnation of affected organs at El-Mahalla El-Kubra abattoir at 7500 USD and 3700 USD in buffaloes and cattle, respectively [170].

The number of bTB infected animals is growing in infected dairy herds in Egypt as recorded recently by Elsohaby et al. who reported a high prevalence ranging from 6% to 66% in blood and 35%–61% in milk samples using PCR, IGRA, and mycobacterial culture [72]. The authors attributed these high percentages to the limited sensitivity of the tests used in bTB eradication programs, which result in persistence of the infection within dairy herds over time. Furthermore, high prevalence of JD in dairy herds in Egypt and the presence of a high proportion of cows in advanced stages of the disease affect the diagnostic performance of bTB tests.

There is a markedly wide geographic variation in the prevalence of bTB in Egypt. Despite the underlying causes of this variation being poorly understood [171], it may be attributed to the diversity of agroecological nature, species, breeds, numbers of reared animals, and types of husbandry systems prevalent in different regions in Egypt. In addition, the greater number of animals slaughtered than normal during religious feasts and other sociocultural events has a role in this variation of prevalence of bTB between studies [171]. Moreover, insufficient numbers of professional meat inspectors for covering all slaughtered animals, lack of technical facilities at most abattoirs, absence of realistic official reports at abattoirs, low offered compensation values, especially in the case of partially condemned organs, and routine work difficulties are limiting factors facing the detection of actual prevalence of bTB at Egyptian abattoirs. The wide illegal slaughtering outside of abattoirs without any veterinary supervision also poses a great health risk to consumers. Further, the widespread mixed-livestock rearing systems, particularly in both pastoralist and smallholder communities, in which several species, such as sheep, goat and even camels were reared with cattle, shared the same pasture and air space and usually kept at night at the low-hygiene and poorly-ventilated farmers houses [43].

## 10. Conclusions

Despite annual efforts, bTB continues to have a huge impact on human and animal health, particularly in developing countries. The absence of a gold standard single test to detect all cases of bTB, the absence of a realistic vaccine against the disease, and the zoonotic impact are the main challenges that should be considered in future research. This review has discussed bTB as a disease, conventional and recent diagnostic methods, and the emergence of MDR-*Mycobacterium* species. Our review is intended to renew interest in considering the emergence of infection caused by drug-resistant *M. bovis* as an important public health threat that menaces the success of TB control programs. 
